# In Situ Raman Measurement of the Growth of SiCOH Thin Film Using Hexamethyl-Disiloxane (HMDSO) Mixture Source in Semiconductor Interconnection

**DOI:** 10.3390/mi16111202

**Published:** 2025-10-23

**Authors:** Hwa Rim Lee, Tae Min Choi, Sung Gyu Pyo

**Affiliations:** School of Integrative Engineering, Chung-Ang University, 84, Heukseok-ro, Dongjak-gu, Seoul 06974, Republic of Korea; ghkfla0725@naver.com (H.R.L.); c79411@gmail.com (T.M.C.)

**Keywords:** real-time, in situ monitoring, Raman spectroscopy, SiCOH thin film, dual laser

## Abstract

This research focuses on the real-time monitoring of SiCOH thin films grown by chemical vapor deposition (CVD) using Raman spectroscopy. To ensure the reliability of CVD-deposited materials in semiconductor processes, the study analyzes the growth and properties of thin films in situ. With the increasing demand for low-dielectric constant (low-k) materials due to the miniaturization of semiconductor components, real-time monitoring becomes essential for controlling film thickness, quality, and composition during deposition. Dual laser wavelengths (405 nm and 532 nm) were used to capture Raman spectra and observe changes in film thickness, crystallinity, and the bonding structures of Si-C, Si-OH, and C-C. The results demonstrate that Raman spectroscopy effectively detects real-time molecular changes in thin films, showing a clear correlation between deposition time and film properties such as crystallinity and bond formation. This approach provides valuable insights for optimizing semiconductor thin film processes in real-time.

## 1. Introduction

The demand for developing low-k materials with high thermal conductivity to accommodate sub-10 nm integrated circuit components has been increasing due to semiconductor miniaturization [1,2]. In particular, CVD-based low-k materials have been widely used for their thermal and mechanical stability, as well as their low-dielectric constant [3,4,5]. SiCOH films have garnered significant attention in recent years due to their compatibility with current semiconductor technologies [6,7,8]. While many research groups have reported the deposition and characterization of SiCOH films as low-k materials [9,10,11,12,13,14,15], studies focusing on in situ analysis are still lacking.

In situ monitoring allows for the precise control of thin film thickness, quality, structure, and composition based on real-time data. Therefore, in situ monitoring is a crucial technique for elucidating thin film growth mechanisms and controlling their properties [16,17]. Moreover, installing analytical equipment in processing tools to monitor raw material conditions before processing, reaction states during deposition, and thin film conditions after deposition in real-time can aid in yield management and contribute to final quality assurance. In this study, in situ Raman spectroscopy was employed to monitor the phenomena from the early stages of thin film formation to the growth stage in real-time. Raman spectroscopy provides comprehensive information about the immediate molecular environment within a sample, regardless of whether the sample is crystalline, amorphous, or liquid [18,19,20]. Indeed, Raman spectroscopy is frequently used to obtain in situ spectral information across a wide range of systems [21,22,23]. Since Raman spectroscopy utilizes short wavelengths, using light from various wavelength bands depending on the material being analyzed can provide more precise information. Raman characteristics and specific peak positions of materials are related to the unique chemical structures of the materials, and the molecular fingerprint remains the same regardless of the excitation wavelength. However, different excitation wavelengths offer distinct strengths and weaknesses, allowing users to optimize the measurement of different samples by selecting appropriate Raman excitation laser wavelengths [24].

In this study, SiCOH thin films deposited via chemical vapor deposition (CVD) using an HMDSO-Ar gas mixture source were analyzed in real-time using Raman spectroscopy with dual laser wavelengths (405 nm and 532 nm). This study utilizes a multi-array Raman analyzer and presents a Raman analysis method optimized for thin films, improving upon conventional techniques. This method provides Raman laser wavelength bands that can be optimized for various thin films.

## 2. Materials and Methods

The SiCOH thin films were deposited on 4-inch p-type Si [(100), 1–20 Ω.cm] wafers by CVD, using a HMDSO-Ar gas mixture (KITECH, Cheonan, Republic of Korea) (flow rate: 40 sccm) as the source. The carrier gas was O_2_ with a flow rate of 25 sccm and the reaction temperature was 300 °C. After 30 min of thin film growth, the finished samples were subjected to post-treatment by depositing thin films at RF power 200 W and a reaction temperature of 300 °C during 0~150 s, and then the changes in the thin films of the five samples (0, 60, 90, 120, 150 s) were analyzed. A total of 5 samples were characterized by XRD (X-ray diffraction) and Raman spectroscopy (Nanobase, Seoul, Republic of Korea) (405 and 532 nm).

**Figure 1:**
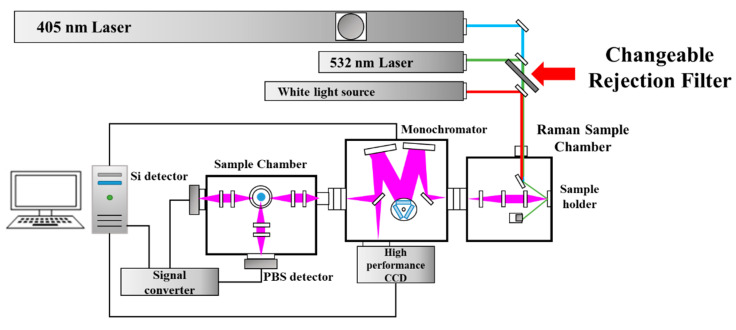
Schematic diagram of Raman spectroscopy.

A multi-array light source Raman spectrometer capable of utilizing two wavelengths simultaneously was employed to perform the measurements. The Raman spectrometer was used with a short wavelength 405 nm laser light source to increase sensitivity and a long wavelength 532 nm laser light source to prevent luminescence. To overcome the shortcomings of the limited analytical sample, which is a shortcoming of the conventional single wavelength spectroscopy, materials such as metal oxides, semiconductor materials, and organic thin films were analyzed. Additionally, Gaussian filtering was used to reduce image noise caused by fluorescence. The possibility of Raman spectroscopy as a real-time process diagnosis that can monitor the internal environment of a process chamber of a semiconductor thin film and the formation of a thin film in a vacuum environment in a process chamber was examined. Raman spectra were first obtained using a laser source of 532 nm excitation wavelength with power ranging from 2 to 30 mW and with a exposure time in the range of 500–5000 ms. A laser source of 405 nm was also used, which is compatible with Raman machines using a 532 nm laser. A simple schematic and Raman instrument specification is shown in Figure 1 and Table 1, respectively.

## 3. Results

**Figure 2:**
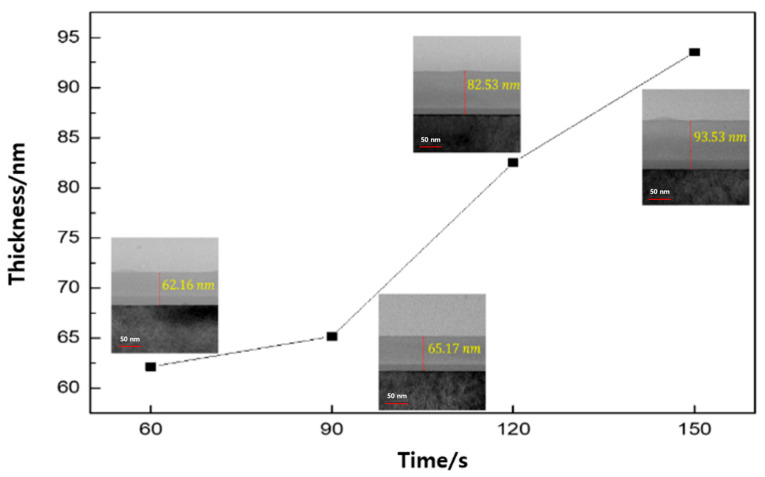
TEM image of SiCOH thin film with different post-treatment time.

Figure 2 shows the cross-sectional TEM image of the SiCOH thin film and its change in thickness. The brightest part is the Si wafer, and the bottom part is the grown thin film. x-axis represents post-processing time, and the y-axis is the thickness of the thin film that is added to the thickness of the thin film produced in the post-treatment process [25]. The film exhibited thicknesses of 62.16 nm at 60 s of post-treatment, 65.17 nm at 90 s, 82.53 nm at 120 s, and 93.53 nm at 150 s, respectively. As seen from Figure 2, as the deposition time increases, the thickness increases, and it can be seen that the film grows rapidly from 120 s.

**Figure 3:**
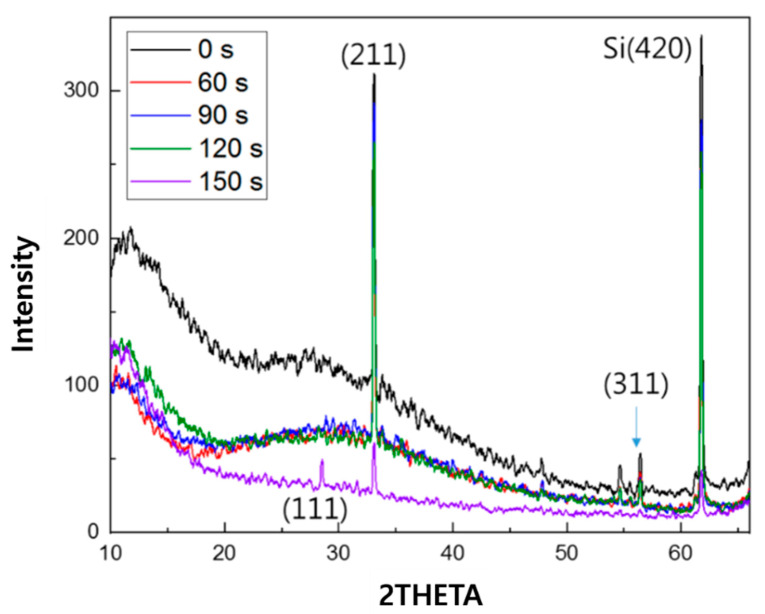
XRD of SiCOH thin film with different post-treatment time.

Figure 3 shows the characteristic silicon peaks in SiCOH samples at post-treatment times of 0 s, 60 s, 90 s, 120 s, and 150 s, indicating crystallinity in the (111), (211), (311), and (420) directions, represented by 2θ values of 28.5°, 33.0°, 56.5°, and 61.8°, respectively. As the post-treatment time increased, the intensity of each peak decreased, indicating a reduction in crystallinity.

To demonstrate the reproducibility of thin film Raman measurements, four SiCOH thin films deposited by the CVD process (post-treatment time 60 s, 90 s, 120 s, and 150 s) were subjected to repeated measurements of the FWHM of the peaks associated with C bonds in the Raman spectra to determine the error range. As a result, the FWHM value used as an index of crystallinity had errors (%) of 1.1, 1.1, 1.4, and 2.1, respectively. This is the average of 20 measurements taken using the Gaussian method and represents the error of the Raman measurement. The respective error as a function of post-processing time is less than 3%, indicating that the reproducibility of thin film Raman measurements is high with minimal error values.

**Figure 4:**
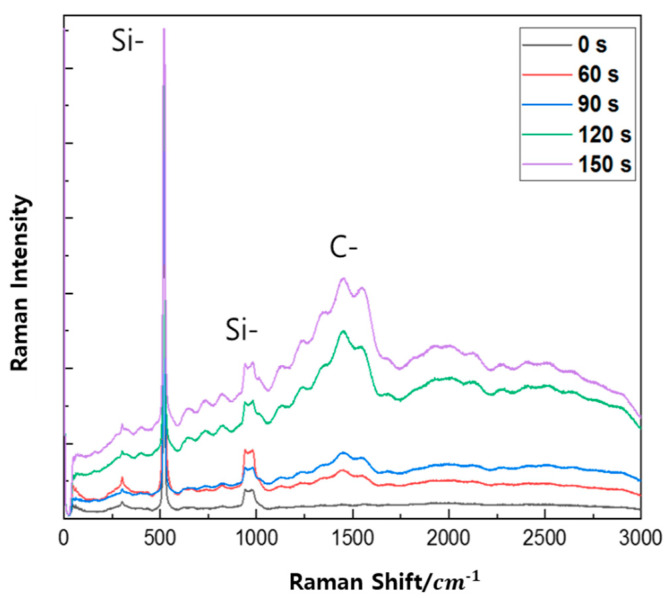
Raman spectroscopy (532 nm) of SiCOH thin film with different post-treatment times.

**Figure 5:**
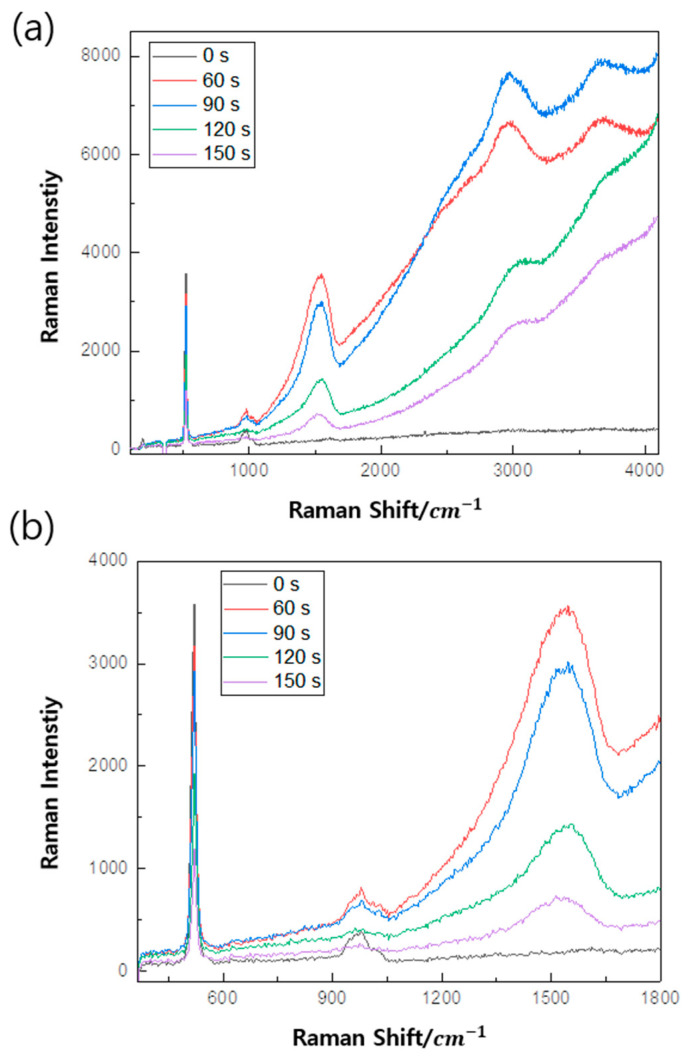
Raman spectroscopy (405 nm) of SiCOH thin film with different post-treatment times. (**a**) A total of 405 Raman spectrum from 100 to 4100 cm^−1^ and (**b**) 370 to 1800 cm^−1^.

**Figure 6:**
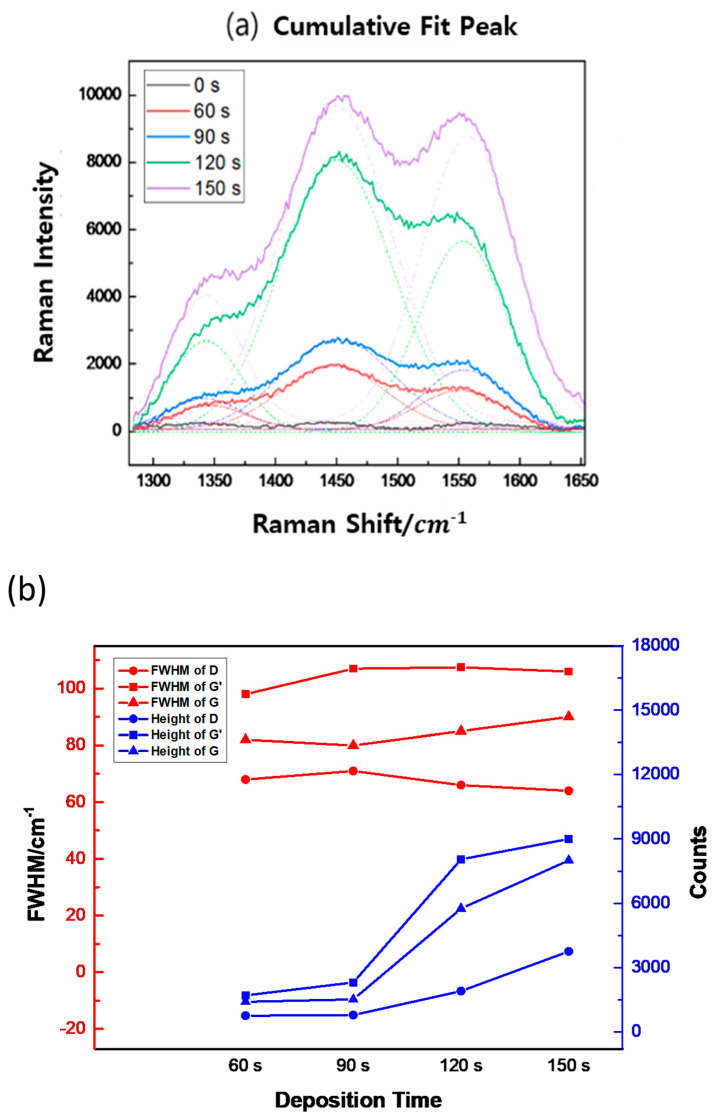
Fit peak of 532 nm laser Raman from 1250 to 1650 cm^−1^. (**a**) Fitting curve of Raman shift value at 1343 cm^−1^ (D), 1449 cm^−1^ (G′), and 1557 cm^−1^ (G (C-H_3_ symmetric bending)). (**b**) Rate of change in each peak.

In order to evaluate the film quality, Raman spectroscopy with 532 nm and 405 nm Raman were carried out and shown in Figure 4 and Figure 5, respectively. To determine crystalline and amorphous states using Raman, the most common method used is comparing peaks at 520 cm^−1^ and 980 cm^−1^. The low energy peak (520 cm^−1^) is caused by the amorphous state but shifts toward higher energy. This is caused by a change in the Raman peak form of the crystalline fraction. Peaks between 500 cm^−1^ and 520 cm^−1^ provides information about the size of the Si crystal, and it corresponds to the Si-Si longitudinal optical (LO) peak (in bulk silicon) and affects the crystalline Raman peak appearing at 520 cm^−1^ [26] due to the transverse acoustic (TA) phone mode of being amorphous. Also, the peaks at 940 cm^−1^ and 980 cm^−1^ corresponded to silanol groups (Si-OH) [27]. These two peaks appear in both Si-based materials. The peaks appear between 1250 cm^−1^ and 1650 cm^−1^ due to amorphous carbon, and they are revealed by the D band, which provides information about defects in the carbon bonding structure, and the G band, which provides information about the carbon bonding structure. A detailed fitting process is shown in Figure 6.

The SiCOH thin film was measured at different Raman lasers. The Si-Si and Si-OH peaks appeared at the same position and the carbon peak intensity appeared higher in the left and right of the 1500 cm^−1^ range, but the D and G peaks did not appear as in 532 Raman. Also, peaks that did not appear in 532 nm in 3000 s appeared in 405 nm.

Compared to 532 Raman, 405 Raman provides more information about SiCOH and Raman signals on Si substrates due to its high resolution, high sensitivity, and small penetration depth. This is also because it can be used for the surface auto resonance phenomenon of Depth of Focus (DOF) [26,28,29].

**Figure 7:**
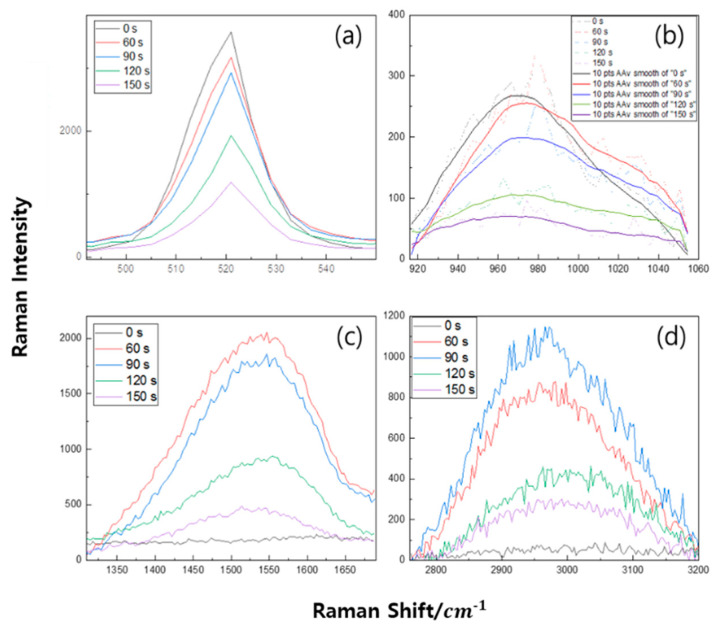
Fit peak of 405 Raman from (**a**) 490 to 550 cm^−1^, (**b**) 910 to 1055 cm^−1^, (**c**) 1300 to 1700 cm^−1^, and (**d**) 2770 to 3200 cm^−1^.

**Figure 8:**
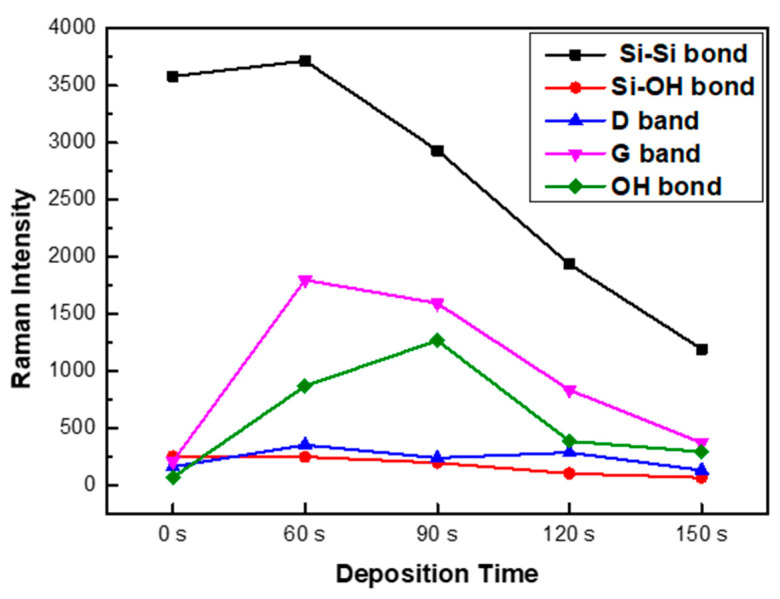
Intensity of 405 Raman as a function of deposition time.

Based on the strong background supported by experiments and data analysis of the Raman spectroscopy of carbon materials, the C-H bond was selected and analyzed in detail. This method is performed after minimizing the crystalline phase analysis error and the thin film concentration analysis error of the deposited film by adjusting the equipment. In order to reduce the influence of a specific peak, which occupies the largest portion of the total peak area of the spectrum, and to perform a fair correlation evaluation on relatively small peaks, the correlation is determined by dividing the entire area into several sections. As seen in Figure 6a, the ratio of the D (1344 cm^−1^) and G (1558 cm^−1^) intensities in the Raman peaks of the deposited thin film is close to 0.5, and the rate of change varies with deposition time, including the G’ peak, which is caused by double Raman scattering and is also known as the 2D band [30,31,32]. A SiCOH thin film with a constant thickness of HMDSO was deposited on a Si wafer according to the growth time, and the phonon modes, LO, and transverse optical (TO) phonon modes were analyzed [33]. The 3C-Si surface deposited on SiO_2_ may exist in the form of amorphous 3C-SiC, including activated carbon. It was demonstrated by Ferrari and Robertson [34] that the C-C bond of graphite is formed when the G peak position is about 1580 cm^−1^ and the intensity I (D)/I (G) ratio increases. This increase occurs because of (i) the amount of “unorganized” carbon in the sample increases and (ii) the size of the graphite crystals decreases. Therefore, as shown in Figure 6b, it can be seen that as the deposition time increases, the C-C bond decreases. In particular, the inclination between 90 s to 120 s is a sharp phenomenon that appears from 90 s, whereby bonding to C is rapidly increased, leading to a rapid increase in thickness. Vas (vibration of asymmetric) of CH_3_ at 2960 cm^−1^ and 4000–2500 cm^−1^: H-X (X = O, N, C, S). Thus, it can be seen that there was a large release of hydrogen between 90 s and 120 s. As shown in Figure 7, as the deposition time increases, the bond intensity between Si-Si bonds decreases. In particular, after 90 s, the Si-bonds are drastically lowered and the C-O-H bonds increase. There was an initial increase in the OH bond (60 s, 90 s) and the thin film deposition time increased to 120 s. The Raman intensity as a function of deposition time is shown in Figure 8.

## 4. Discussion

In this study, in situ Raman spectroscopy was used to monitor the phenomena from the initial stages of thin film formation to growth in real-time. By introducing a multi-array Raman analyzer, it is of great significance that a Raman analysis method optimized for thin films, rather than general Raman analysis, has been proposed. This method provides a Raman laser wavelength band that can be optimized for various thin films. The growth rate of the thin film and the crystallinity of the thin film shown in Figure 2 and Figure 3 can be confirmed by XRD or TEM. This is because real-time in-chamber analysis was not performed, and the obtained results may not fully represent the actual phenomena occurring inside the reactor. It can be seen that the Raman shift appears different in the laser wavelength band depending on the deposition time of the SiCOH low-k thin film. This implies that selecting the Raman laser wavelength range optimized for various thin films is essential to accurately understand the thin film growth process. In addition, crystallinity affects the sharpness and intensity of Raman peaks. This study demonstrated that the crystallinity of SiCOH thin films could be monitored by evaluating the FWHM of specific Raman peaks. A lower FWHM value indicates higher crystallinity [35,36]. The FWHM of G after 90 s showed an increasing trend, which is thought to be due to the effect of SiCOH film thickness. By analyzing the FWHM values over time, the crystallinity of the thin film during deposition can be inferred, allowing for adjustments to optimize the thin film properties in real-time. In the case of the SiCOH thin film, meaningful results were found in the 405 nm laser Raman spectra and the 532 nm laser Raman spectra (Figure 5, Figure 6 and Figure 7). In particular, as seen in the 532 nm laser Raman spectra, it was found through real-time analysis that the D/G ratio changes rapidly as the deposition time increases. In addition, strong C-C bonds and Si-C bonds were strongly formed at the beginning of the reaction, but as the deposition time increased, the formation of C-O-H bonds increased. Notably, the rapid increase in film thickness after 90 s is related to the increase in C-O-H bonds. And it was found that the incubation time of the SiCOH thin film was related to the C-C bond and the Si-C bond at the beginning of the reaction. Changes in crystal structure inferred from changes in Raman spectra can be confirmed using other characterization techniques in addition to the XRD analysis discussed in this study. Kruchinin et al. demonstrated the presence of Si-Si bonds in SiCOH low-k dielectric films grown on Si substrates via PECVD by XPS, FTIR, and Raman spectroscopy analysis [15].

While this paper primarily focused on the structural and compositional analysis of SiCOH thin films, the information obtained from Raman spectroscopy can be correlated with dielectric properties. Changes in the chemical composition and crystallinity, as detected by Raman spectroscopy, influence the dielectric constant of the material. For example, the increase in Si-OH bond concentrations with deposition time, as shown in Raman spectra, can affect the dielectric properties of the films. By correlating these spectral changes with dielectric measurements from capacitance–voltage profiling, Raman spectroscopy can indirectly monitor changes in the dielectric constant [37].

## 5. Conclusions

In this paper, to ensure the reliability of CVD materials for semiconductor thin film processes, SiCOH thin films grown by CVD were analyzed as a function of post-processing time by Raman spectroscopy using a dual laser (405, 532 nm). The results indicated a clear correlation between deposition time and film properties, such as thickness and crystallinity. The 532 nm laser Raman spectrum showed that the D/G ratio changed rapidly as the post-processing time increased, confirmed by real-time analysis. Compared to 532 Raman, 405 Raman provides more information about SiCOH and Raman signals on Si substrates. After 90 s of deposition, C-O-H bonds were rapidly formed, accompanied by a sharp increase in film thickness. The dual laser approach provided complementary information.

These results confirm that Raman spectroscopy is an effective tool for in situ monitoring of thin films, enabling precise control of film growth and properties in real-time. This method allows for adjustments during deposition, optimizing film quality and consistency. The findings highlight the potential of this approach in improving the reliability of CVD processes in semiconductor manufacturing, particularly for low-k materials required in the miniaturization of semiconductor devices.

## Figures and Tables

**Table 1:** 405, 532 Raman instrument specification.

	405 nm	532 nm
Light Source	405 nm laser, 30 mW	532 nm DPSS laser, 100 mW
Raman Filter Set	Grade 4: 200 cm^−1^ (150 cm^−1^ typ.)	Grade 3: 70 cm^−1^ (50 cm^−1^ typ.)
CCD	-ICX674 Camera (Sony, Tokyo, Japan) -1932 × 1452 pixels	-ICX674 Camera -1932 × 1452 pixels
Grating	2400 lpmm@532 nm grating	2400 lpmm@532 nm grating

## Data Availability

Dataset available on request from the authors.

## Abbreviations

The following abbreviations are used in this manuscript:

HMDSO	Hexamethyl-disiloxane
CVD	Chemical vapor deposition
XRD	X-ray diffraction
LO	Longitudinal optical
TA	Transverse acoustic
DOF	Depth of Focus
TO	Transverse optical
